# Understanding the phases of vaccine hesitancy during the COVID-19 pandemic

**DOI:** 10.1186/s13584-022-00527-8

**Published:** 2022-03-22

**Authors:** Dewesh Kumar, Mansi Mathur, Nitesh Kumar, Rishabh Kumar Rana, Rahul Chandra Tiwary, Pankaja Ravi Raghav, Amarendra Kumar, Neelesh Kapoor, Medha Mathur, Tanya Tanu, Soumitra Sethia, Chandrakant Lahariya

**Affiliations:** 1 Department of PSM, Rajendra Institute of Medical Sciences, Ranchi, Jharkhand 834001 India; 2 Immunization Technical Support Unit, Ministry of Health and Family Welfare, New Delhi, India; 3 Department of Community Medicine, Subharti Medical College, Meerut, U.P. India; 4 Department of PSM, SNMMC, Dhanbad, Jharkhand India; 5 Department of Community Medicine, Index Medical College, Indore, M.P. India; 6 Department of Community and Family Medicine, AIIMS, Jodhpur, Rajasthan India; 7 Subregional Team Leader Office, WHO Country Office for India, Ranchi, Jharkhand India; 8 Orbi Health, New Delhi, Delhi India; 9 Department of Community Medicine, Geetanjali Medical College and Hospital, Udaipur, Rajasthan India; 10 Department of Community Medicine, Government Medical College, Khandwa, M.P. India; 11 Foundation for People-Centric-Health Systems, New Delhi, India

**Keywords:** COVID 19 pandemic, COVID vaccine, Vaccine hesitancy, Vaccine eagerness, Vaccine ignorance, Vaccine resistance, Vaccine confidence, Vaccine complacency, Vaccine apathy

## Abstract

Vaccine hesitancy is an important feature of every vaccination and COVID-19 vaccination is not an exception. During the COVID-19 pandemic, vaccine hesitancy has exhibited different phases and has shown both temporal and spatial variation in these phases. This has likely arisen due to varied socio-behavioural characteristics of humans and their response towards COVID 19 pandemic and its vaccination strategies. This commentary highlights that there are multiple phases of vaccine hesitancy: Vaccine Eagerness, Vaccine Ignorance, Vaccine Resistance, Vaccine Confidence, Vaccine Complacency and Vaccine Apathy. Though the phases seem to be sequential, they may co-exist at the same time in different regions and at different times in the same region. This may be attributed to several factors influencing the phases of vaccine hesitancy. The complexities of the societal reactions need to be understood in full to be addressed better. There is a dire need of different strategies of communication to deal with the various nuances of all of the phases. To address of vaccine hesitancy, an understanding of the societal reactions leading to various phases of vaccine hesitancy is of utmost importance.

## Background

The deluge of COVID patients has overwhelmed healthcare systems all over the world. The only hope to controlCOVID-19 during the pandemic was earnest research on developing and producing vaccines against COVID-19.

Whenever there is vaccine introduction, vaccine hesitancy also comes into play due to the complex interaction of vaccine related factors, vaccinees’ dynamics, and other external issues [1]. The phenomenon of vaccine hesitancy existed long before the current pandemic and has recently been considered as one of the top ten threats to health by the World Health Organization [2]. The 3 “Cs” that inadvertently or otherwise influence vaccine acceptance are complacency, convenience and confidence [3]. In due course of time, some experts have tried to develop a measuring tool for assessing vaccine hesitancy in terms of 5C psychological antecedents of vaccination: confidence, complacency, convenience (constraints), calculation, and collective responsibility [4]. Stimulating the acceptance of vaccines against COVID-19 requires the comprehensive understanding of the reasons of willingness and related concerns of people and the trustworthy sources of information in their decision-making.

In relation to the COVID-19 pandemic, the world has also faced the unprecedented public health crisis of an infodemic, too much information including false information; and this has negatively impacted good decisions pertaining to health [5]. The spread of misinformation was not limited to disease transmission, but also accompanied the COVID vaccine roll out [6].

## Main text

### Different phases of vaccine hesitancy

Since the emergence of COVID 19 pandemic, there have been numerous studies on vaccine hesitancy related to COVID 19 vaccine [7–15]. Understanding of vaccine hesitancy has been different by experts in different parts of world. This has led to a diverse array of definitions [16, 17]. There is a need to understand this phenomenon as a behavioural characteristic which changes according to human mental capacity and thoughtfulness. Considering this, the very existence of vaccine hesitancy in an individual, family, community and global is dynamic and very complex.

**Fig. 1:**
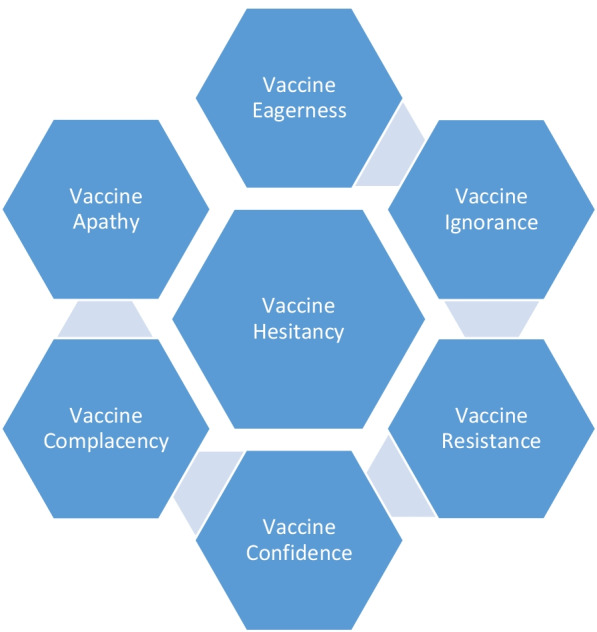
Various phases of Vaccine hesitancy during COVID 19 pandemic. There are multiple phases of vaccine hesitancy: Vaccine Eagerness, Vaccine Ignorance, Vaccine Resistance, Vaccine Confidence, Vaccine Complacency and Vaccine Apathy. These are societal reactions observed or expected to be observed during various periods of COVID 19 pandemic in different regions of world

It has been observed that the nature of vaccine hesitancy changes over time in the same population; and it changes as people experience subsequent vaccination [18]. In this paper, we describe various phases of vaccine hesitancy (Fig. 1) and how vaccine hesitancy has changed during the COVID 19 pandemic.

### Vaccine eagerness/desperation

During the early stages of COVID pandemic, when there was great fear of mortality and morbidity due to SARS COV-2 virus amongst the general population, significant keenness towards vaccines amongst the public was observed [19, 20]. Most people all over the world were eagerly waiting for COVID 19 vaccine in order to get rid of the strict lockdowns and resume their normal life [21]. At that stage, the public was aware that even after the introduction of vaccine, they might have to wait to get vaccine access based on the prioritization criteria. This was because of limited vaccine availability in the initial phase and it took some time before it was made available widely. The waiting period for some sections of people in the society made them more desperate for vaccines. This was also seen amongst the parents who were more concerned about their children when there was no COVID 19 vaccines approved for children. During this phase of eagerness, a vast number of vaccine trials all over the world were conducted to test the efficacy and safety of the various vaccine candidates [22]. This societal reaction may not be considered as part of vaccine hesitancy by some experts but was an important phase encountered during this pandemic.

### Vaccine ignorance

Ignorance may seem like bliss; but sometimes ignorance can be deadly. The United States has a process for Emergency Use Authorization (EUA) of new drugs and biologics. It is faster than full licensure but considered temporary [23]. The US Food and Drug Administration has issued EUAs for COVID-19 vaccines, but ignorance about the EUA of COVID 19 vaccines and the effectiveness of vaccine has led to some untoward results regarding acceptance of these vaccines all over the world. After the EUA of COVID-19 vaccines, people out of their ignorance started questioning the vaccine development, its efficacy, safety, and appropriateness for roll out in the general population [9, 24]. And moreover, none of the experts or researchers were aware of the effectiveness of the vaccine candidates against the then prevalent strains and subsequent mutant strains in future [22]. Some health experts also raised their eyebrows over the vaccine roll out after the EUA which prevented a major chunk of population from getting vaccinated. Either they were unaware of EUA and its procedural mechanism or they may not have been convinced with the EUA for COVID vaccines, as it was never done before. With the ignorance and confusion over COVID 19 vaccines roll out, people perceived themselves as guinea pigs for the COVID 19 vaccine drive by the government agencies [25]. The ignorance phase was inevitable since even some health care providers were not fully confident about the vaccine’s safety and efficacy.

Another aspect of hesitancy due to ignorance followed the lowering of preventive measures by vaccinated and even unvaccinated people under the misconception that the vaccine was the complete solution to COVID-19. People also were unaware of the fact that after partial or even a full course of vaccination, they may contract infection but the severity would be less. This was the phase when the concept of break-through infections was not common. These factors led to a rising incidence in infections due to increased transmission of COVID 19 virus in the community. This ignorance also fuelled vaccine hesitancy in the communities and gave antivaxxers material to propagate their agenda [26].

### Vaccine resistance

Intertwining with the phase of vaccine ignorance, the phase of vaccine resistance also showed its presence during the COVID 19 pandemic. Amidst bizarre theories of staunch anti-vaxxers, reduction in number of severe cases and reporting of Adverse Events Following Immunization (AEFI) led to this phase of vaccine hesitancy. Though most of the AEFI were mild, a few rare ones were serious or severe. Arzarpaneh and colleagues associated 15 types of cognitive bias as possible contributors to vaccine hesitancy [9].

In this phase, social media was rife with posts disparaging the vaccines [27]. The theories of antivaccine supporters spreading the infodemic online through various social media platforms resulted in vaccine resistance in various communities [9, 28]. The anti-vaccine movement swept through privileged as well as under privileged communities all over the world. The rise of anti-vaccine movements may correspond with different characteristics of members of the population such as unawareness, anxiety and religious beliefs [26].

### Vaccine confidence

This phase occurs when the morbidity and mortality due to an infection such as COVID-19 is primarily seen in unvaccinated individuals. It suggests to people that the vaccine is effective against the disease particularly in reducing hospitalizations and deaths [29]. In this phase, those who were hesitant initially due to their concern regarding vaccine novelty, composition and the processes of manufacture and delivery become vaccinated either for professional or personal reasons. As per protection motivation theory (PMT) and the health belief model, protective health behaviours, such as COVID-19 vaccination, will be adopted in due course of time if the individual rationally assesses threat to be severe with a high probability of occurrence [28, 30], and the individual perceives the overall benefits to exceed the risks and costs. Most countries have undergone severe waves of the pandemic and in most countries COVID 19 vaccines are either free or affordable to most sections of society. The combination of these conditions increased confidence in getting the vaccine during this phase.

### Vaccine complacency

Vaccine complacency is a recurring impediment to vaccination [31]. With respect to COVID 19 vaccination coverage, this occurs during the period when there is a low rate of transmission and illness between the waves of the pandemic. During this phase, although people may have taken no dose or just a first dose of the vaccine, complacency prevents them from getting fully vaccinated. This continues to make the public as a whole vulnerable to COVID 19 due to inadequate population immunity against the virus and it also increases the risk of development of new mutant strains.

There have been instances of vaccine complacency in the past against other vaccination drives [8]. Vaccine complacency for the same vaccine differs from region to region at the same or different periods of time depending on socio-behavioural factors. This temporo-spatial variation of vaccine complacency occurs with COVID 19 too. During COVID-19, there has also been a much lower occurrence of influenza across the world. Although higher influenza immunization rates were seen in some places [31], use of masks and social distancing probably also played a major role in this phenomenon. Experts fear that this may lead to complacency towards periodic influenza vaccination in the coming seasons [32, 33]. However, while the benefits of COVID 19 vaccination are distinct and well acknowledged by international agencies, vaccine complacency continues to reduce the prospective assistances of vaccination at population level in combating this pandemic. This may also arise when milder strains are in circulation and people become complacent to vaccination.

### Vaccine apathy

Although only a few experts describe vaccine apathy as different from vaccine hesitancy [34], we consider vaccine apathy as a component of vaccine hesitancy. Vaccine apathy is defined as disinterest or feeling of not being interested in vaccination. It is characterized by weak attitudes towards getting vaccinated and associated with little time spent considering vaccination [35]. This may occur distinctly, or it may co-exist with complacency or other phases of vaccine hesitancy in the community.

Vaccine apathy occurs across various socioeconomic groups. For some, vaccination may be low-priority due to loosening of COVID-19 restrictions and return of life back to normal; whereas some individuals may be overwhelmed with higher priority problems of daily earning and other social responsibilities. Determining the magnitude of the vaccine-apathetic population is challenging. It is difficult to predict, like voters’ turnout in polls. Another problem in determining the true magnitude of the vaccine-apathetic population is that people tend to want to present themselves in a manner they think is favourable, so-called social desirability bias [36]. Hence apathetic people may describe their disinterest in personal terms, e.g. lack of concern, indifference to preventive health care, health disinterest/fatalism, rather than in terms related directly to vaccine efficacy and safety, and issues with the clinical trials.

Persons with antivaccine positions differ from apathetic people. The former have strongly held and highly defended attitudes, whereas in comparison the latter have weakly negative attitudes toward vaccines. Therefore, effective communication strategies to influence these two groups differ markedly. In the apathy phase of vaccine hesitancy, vaccine promotion approaches specifically addressing the apathetic population may be a vital component in increasing the vaccination coverage and for achieving the national vaccination goals.

## Conclusions

Though the phases of vaccine hesitancy have been described in sequential manner in this article, various phases can co-exist at the same time in different regions or even in the same region. Several factors leading to various societal reactions can influence the phases of vaccine hesitancy with respect to stage of the pandemic and geographical location. Various solutions to the problem of vaccine hesitancy have been proposed, but the comprehension of its complexity and the origin, existence and spread of each of its types or phases is essential to address it more convincingly [36–38]. Humanity is in dire need of a solution for vaccine hesitancy, and we believe that the solution lies ineffective communication and appropriate mass education. The communication strategies for each phase need to be planned and implemented meticulously. We may learn from the experiences of Israel’s successful COVID vaccination program in addressing vaccine hesitancy and achieving a very high coverage rate during the first three months of the initiation of COVID 19 vaccination [38]. There are many countries which have handled vaccine hesitancy and rolled out the vaccination program successfully with effective communication. Learning from different parts of world in dealing various phases of vaccine hesitancy may help each country make effective communication plans for individuals, families, communities, and the nation as a whole.

The societal reaction to the vaccine inequity in regards to vaccine availability in different parts of world may also lead to vaccine hesitancy which has not been discussed here and is a limitation of this paper. In addition, COVID-19 vaccination has been primarily an issue of adult immunization. In low and middle income countries, adult immunization is in its infancy [39]. Therefore, successful approaches for addressing vaccine hesitancy that are developed and implemented during the COVID-19 pandemic might be helpful in shaping the future direction of adult vaccination in the world.

## Data Availability

Not applicable.

## Abbreviations

AEFI: Adverse Event Following Immunization
EUA: Emergency Use Authorization
PMT: Protection motivation theory
